# Pentacyclic Triterpenes in *Euphorbia microsciadia* with Their T-cell Proliferation Activity

**Published:** 2011

**Authors:** Abdul Majid Ayatollahi, Mustafa Ghanadian, Suleiman Afsharypour, Omer Mohamed Abdella, Mehdi Mirzai, Gholamreza Askari

**Affiliations:** a *Phytochemistry Research Center, Shahid Beheshti University of Medical Sciences, Tehran, Iran. *; b *Isfahan Pharmaceutical Sciences Research Center, Isfahan University of Medical Sciences, Isfahan, Iran.*; c *International Center for Chemical and Biological Sciences, Panjwani Center for Molecular Medicine and Drug Research, University of Karachi, Karachi, Pakistan. *; d *Department of Nano technology Research Center, Shahid Beheshti University of Medical Sciences, Tehran, Iran.*; e *Faculty of Health, Isfahan University of Medical Sciences, Isfahan, Iran. *; f *Department of Pharmacognosy, Shool of Pharmacy and Pharmaceutical Sciences, Shahid Beheshti University of Medical Sciences, Tehran, Iran.*

**Keywords:** *Euphorbia microsciadia*, Oleane, ursane and lupane pentacyclic triterpenes, Triterpene mass fragmentation pattern, T-Cell proliferation assay

## Abstract

The ethyl acetate partition of dried methanolic extract of aerial parts of *Euphorbia microsciadia* (Euphorbiaceae) afforded three pentacyclic triterpenes, betulinic acid (1) from lupane type, oleanolic acid (2) from oleane type and ursolic acid (3) from ursane type triterpenes that are reported for the first time in this plant. These three compounds were structurally compared through their mass fragmentation pattern, nuclear magnetic resonance (NMR) data and their biologic immunomodulatory effects. The structures of the isolated compounds were elucidated by ^13^C- and ^1^H-NMR as well as 2D-NMR, IR and by the aid of mass fragmentation pattern and comparing with the literature. After running T-Cell proliferation assay, oleanolic acid stimulated proliferation of T-Cells at lower concentration 0.5 µg/mL, while betulinic acid and ursolic acid showed inhibitory activity against T-Cell proliferation with IC_50_- value > 50 µg/mL and 3.01 ± 0.47 µg/mL, respectively.

## Introduction

Euphorbiaceae is one of the largest families of the phylum Anthophyta. In this family, the largest genus is Euphorbia which comprises well over 2000 species in tropical and temperate zones of Asia and other parts of the world. In Iran, 70 species are reported out of which 17 are endemic (1). In traditional medicine, Euphorbia was used as the treatment of intestinal parasites, gonorrhea, wart cures and also in the treatment of skin diseases (2). However, multidisciplinary biological screening tests carried out in recent years have shown that some of them were useful as anti-tumors, pesticides and antiviruses. 

In this research, three triterpenes of three different common pentacyclic classes were isolated from the aerial parts of *Euphorbia microsciadia* (Euphorbiaceae) which is a perennial plant that grows in some parts of Iran (3); there has been efforts to elucidate themthrough Nuclear magnetic resonance (NMR) and other spectroscopic methods, especially the mass fragmentation pattern.

**Figure 1:**
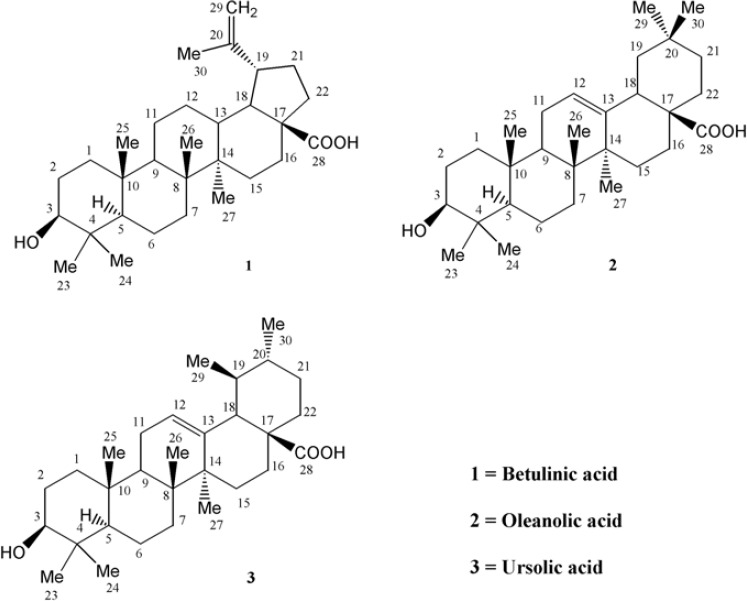
Pentacyclic triterpenes from *Euphorbia microsciadia *(1-3).

## Experimental

*General*

The ^1^H-NMR spectra were recorded on a Bruker Avance AV 300 but ^13^C-NMR and 2D-NMR spectra were recorded on a Bruker Avance AV 600 NMR instrument, using CDCl3 as solvent. The IR spectra were recorded on a JASCO 302-A spectrophotometer and the mass spectra (EI and HREI-MS) were measured in an electron impact mode on Varian MAT 112 or MAT 312 spectrometers. Fast atom bombardments (FAB) MS were measured on Jeol HX110 mass spectrometer.

*Chromatographic conditions*

HPLC: LC-908 Recycling Preparative HPLC Hitachi company (Japan), Column: Jaigel-2H and 1H columns (600 × 20). Column chromatography run by Merck, Silica gel, 63-200µm, flash chromatography on LiChroprep® Si 60 (25-40 µm), Reverse column chromatography by LiChroprep RP-18, 40-60 μm (Merck) and size exclusion chromatography by Sephadex® LH-20 from Sigma-Aldrich. Thin-layer chromatography (TLC) was performed on precoated silica gel GF-254 plates, 20 × 20 cm, 0.5 mm thick (Merck). The visualization of TLC plates was achieved at 254 and 366 nm and cerium sulphate spray reagent 10% H_2_SO_4_ was used for detection. 

*Plant material*

The aerial parts of flowering plant of *Euphorbia microsciadia* Boiss (Euphorbiaceae) were collected in August 2007 from populations growing in Galil-e-Shirvan (near the Turkmenistan border), Northern Khorasan province in Iran. The plant was identified by Mrs. Yasamin Naseh, plant taxonomist (Department of botany, herbaceous sciences research center at Ferdowsi University of Mashhad). A voucher specimen (NO. 2023) of the plant was deposited in the herbarium of the Pharmacognosy department, Faculty of Pharmacy and Pharmaceutical Sciences at Isfahan University of Medical Sciences (Iran).

*Extraction and isolation*

The dried plant (4.5 Kg) was extracted with MeOH (18 L) at room temperature for four days by three times and the resulting extract was concentrated to a 400 g dark green gum. This gum was partitioned into *n*- heptane and aqueous MeOH (80%). The defatted MeOH extract was evaporated and dissolved in H_2_O. The aqueous extract was partitioned into H2O and ethyl acetate (EtOAc). Thereafter, EtOAc part was subjected on RP-18 column chromatography eluting with aqueous methanol 30%, 60% and 100% respectively. The fraction obtained on elution with H2O : MeOH (40 : 60) transferred on another column using Hexane/EtOAc (0→50%). Later, the Hexane : EtOAc (80 : 20) part was separated from the rich chlorophyll content and pigments by size exclusion chromatography on sephadex (LH-20) using Dichloromethane (DCM) / MeOH (1 : 2). Finally, fractions which showed violet spots on TLC with cerium sulphate reagent were selected and purified on Recycling HPLC Jaigel-2H and 1H columns with chloroform as the mobile phase to yield three pure compounds (1-3) that were crystallized in DCM : MEOH (1 : 3).

*T-Cell proliferation assay*

 Fresh venous blood from a normal healthy volunteer was collected in heparinized container and mixed with equal volume of RPMI-1640 incomplete media containing L-glutamine (Mediatech Inc., Herndon, VA, U.S.A.), then layered onto lymphocyte separation medium (LSM) (from MP Biomedicals, Inc., France) and centrifuged at 400×g for 20 min at 25°C. The mononuclear cell buffy coat was removed and cells were washed with incomplete RPMI-1640 and centrifuged for 10 min at 4°C and 300×g. The peripheral blood mononuclear cells (PBMNCs) were resuspended in supplemented RPMI-1640 at 2.5×10^6^ Cells/mL containing 10% fetal bovine serum (FBS) from PAA laboratories GmbH (Pasching, Austria). In a 96-well round-bottomed microtiter plate, 50 µL of cell suspension plus 50 µL of phytohemagglutinin (PHA) with a final dilution of 5 µg/mL, 50 µL supplemented RPMI-1640 and 50 µL of test compounds in a final concentration of 0.5, 5 and 50 µg/mL were added. Cells were cultured at 37°C in a humidified atmosphere of 5% CO_2_ in air for 72 h. Further incubation for 18 h after the addition of thymidine [^3^H] (Amersham, Buckinghamshire, UK) was done and cells were harvested using cell harvester(Inotech Dottikon, Switzerland)and the incorporation was measured by a liquidscintillation counter (Beckman coulter, LS 6500, Fullerton, CA, USA) (4).

*Statistical analysis*

The IC_50_- values were calculated using an Excel based program and reported as mean ± standard deviation (SD) of the mean. Significance was attributed to p-values (p < 0.05) and the probability values obtained by the student t-test between the sample and control data.

## Results and Discussion

Compound 1 was obtained as colorless needles with molecular formula of C_30_H_50_O (calculated: 426.7194) on the basis of positive electron ionization high-resolution mass spectrometry (EI-HRMS) of the molecular ion peak at *m****/****z* 456.3587 matched with the number of carbons and hydrogens counted in NMR data (Table 1).

**Table 1:** ^1^H-NMR and ^13^C-NMR data for the pentacyclic triterpenes in *Euphorbia* *microsciadia*^a^

**C**	**1**	**2**	**3**
	^13^ **C- NMR**	^13^ **C- NMR**	^13^ **C- NMR**
1	38.6 t	39.1 t	38.78 t
2	27.0 t	27.9 t	23.4 t
3	78.7 d	79.7 d	79.0 d
4	38.7 s	40.0 s	39.5 s
5	55.3 d	55.9 d	55.2 d
6a	18.2 t	19.1 t	18.2 t
7a	34.2 t	33.4 t	33.0 t
8	40.5 s	40.2 s	39.0 s
9	50.4 d	48.32 d	47.6 d
10	37.0 s	37.8 s	36.6 s
11	20.8 t	24.0 t	23.7 t
12	25.4 t	123.3 d	125.7 d
13	38.2 d	143.3 s	137.9 s
14	42.3 s	42.4 s	41.9 s
15	30.5 t	28.4 t	29.3 t
16	32.1 t	24.1 t	23.3 t
17	56.1 s	46.6 s	47.9 s
18	49.1 d	41.9 d	52.7 d
19	46.9 d	46.6 t	30.6 d
20	150.6 s	30.4 s	30.3 d
21	37.0 t	34.5 t	27.2 t
22	29.6 t	31.3 t	37.0 t
23	27.5 q	28.8 q	23.4 q
24	15.2 q	16.2 q	16.9 q
25	15.8 q	16.0 q	16.9 q
26	16.0 q	17.7 q	15.4 q
27	14.5 q	26.6 q	24.2 q
28	189.1 s	172.0 s	175.9 q
29	106.4 t	33.7 q	21.0 q
30	19.2 q	24.2 q	23.3 q

Chemical shifts are given in ppm

**Figure 2:**
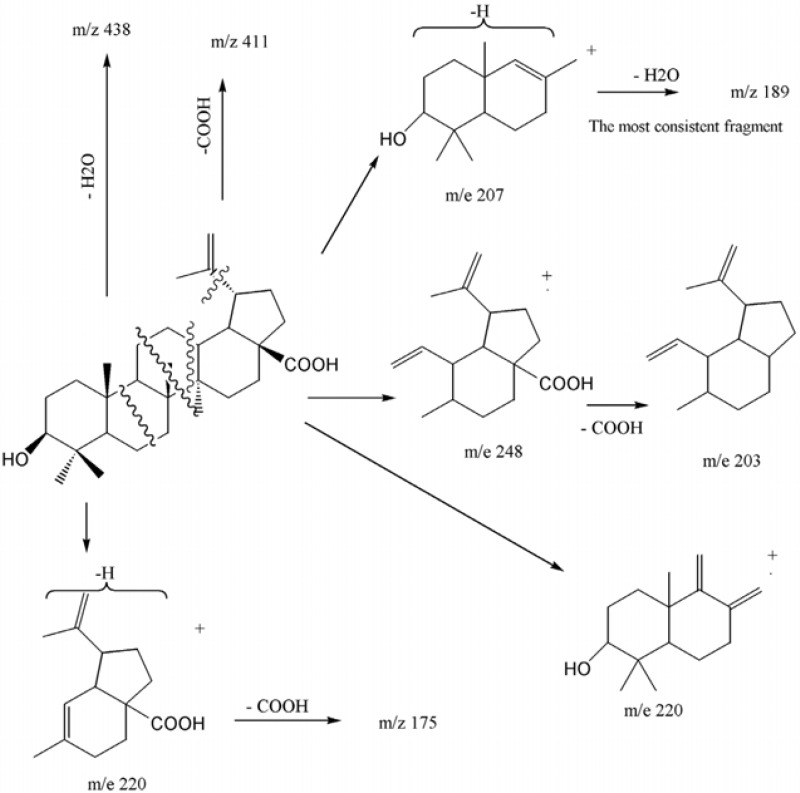
EI-Mass fragmentation pattern of Betulinic acid

With regard to the seven degrees of unsaturation, ^13^C-NMR and Distortionless Enhancement by Polarization Transfer (DEPT) spectral data, five rings, one acidic carbonyl and one double bond were detected in the molecule. IR spectrum showed a broad peak of hydroxyl group at 3446 cm^-1^, prominent carbonyl absorption at 1685 cm^-1^, double bonded peaks at 1645 and 1604, and C-O functionalities at 1236, 1107 and 1034 cm^-1^. ^1^H-NMR revealed six singlet methyls at δ_H_ of 1.67 s, 0.95 s, 0.94 s, 0.91 s, 0.80 s and 0.73 s, a pair of olefinic protons at δ_H_ of 4.71 and 4.58 (each one H, br-s) characteristic of exocyclic methylene group, a carbinolic proton at δ_H _of 3.17 (dd, *J*_ax,ax_ = 10.8, *J*_ax,eq_ 5.1 Hz, H_3_) referring to its axial and α orientation (5), δ_H_ of 2.95 (dt, *J*= 11.1, 3 Hz, H_19_), 2.24 (br-d, *J *= 12.3 Hz, H_16a_), 2.16 (br-t, *J* = 10.4 Hz, H_13_) and 1.95 (dd, *J* = 11.1,4.5 Hz, H_21a_) which were characteristic for lupanetriterpenes (5). Further information about the compound was obtained from typical EI-Mass related to the fragmentation pattern of lupane type triterpenes (Figure 2) through the presence of *m/z *438 [M-H_2_O], 411 [M-COOH], 248 [C_16_H_24_O_2_], 203 [248 - COOH], 220 [C_15_H_24_O], 203 [220 - OH], 220 [C_14_H_20_O_2_], 175 [220 - COOH], 207 [C_14_H_23_O], 189 [207 - H_2_O] 205 and 207 [M - C_16_H_27_] characteristic series for betulinic acid (6).

^13^C-NMR (BB and DEPT) spectra showed thirty carbons comprised of seven methyls, eleven methylenes_, _six methines and six quaternary carbons. Based on above observations, the’ spectral data of compound 1 was similar to those previously reported of betulinic acid (5, 6), except for the assignment of five-member ring carbons. Therefore, the authors decided to assign it again by Double-Quantum Filtered Correlation Spectroscopy (DQF-COSY) and Heteronuclear Multiple Bond Coherence (HMBC) data (Figure 4). The DQF-COSY spectrum indicated following spin systems of correlated protons: (S_1_) -CH_2_-CH_2_-CHO with δ_H _0.88 (br-d, J = 10.4, H_1b_); 1.59 (m, H_2a_); 3.17 (dd, J = 10.8, 5.1 Hz, H_3_), (S_2_)-CH-CH_2_- with δ_H _0.66 (br-d, J = 8.8 Hz, H_5_); 1.46 (m, H_6a_) and (S_3_) CH_2_-CH_2_-CH-CH-CH-CH_2_-CH_2_ with δ_H _1.38 (m, H_11a_), 1.00(br, H_12b_); 2.16 (br-t, J = 10.4 Hz, H_13_); 1.58 (m, H_18_); 2.98 (dt, J = 11.1, 3 Hz, H_19_); 1.95 (dd, J = 11.1,4.5 Hz, H_21a_) and 1.17 (dt, H_22b_). Fragment S_1_, due to its attachment to hydroxyl group, proposed to be C_1_-C_2_-C_3,_ HMBCs of H_5_ with C_1_ confirmed S_2_ as C_5_-C_6 _and HMBCs of δ_H _2.36 (H_19_) with quaternary exocyclic olefinic carbon δ_c _150.62 (C_20_), assigned substructure S_3_ as C_11_-C_12_-C_13_-C_18 _–C_19_-C_21_-C_22_ and δ_c _ = 150.6 as C_20_. The position of six singlet methyls, Me23, Me24, Me25, M26, Me_27_ and Me30 bonded to quaternary carbons C_4_, C_8_, C_10_, C_14_ and C_20 _were determined by their HMBC correlations with substructures S_1_, S_2_ and S_3_ as is shown in Figure. 4.

**Figure 3:**
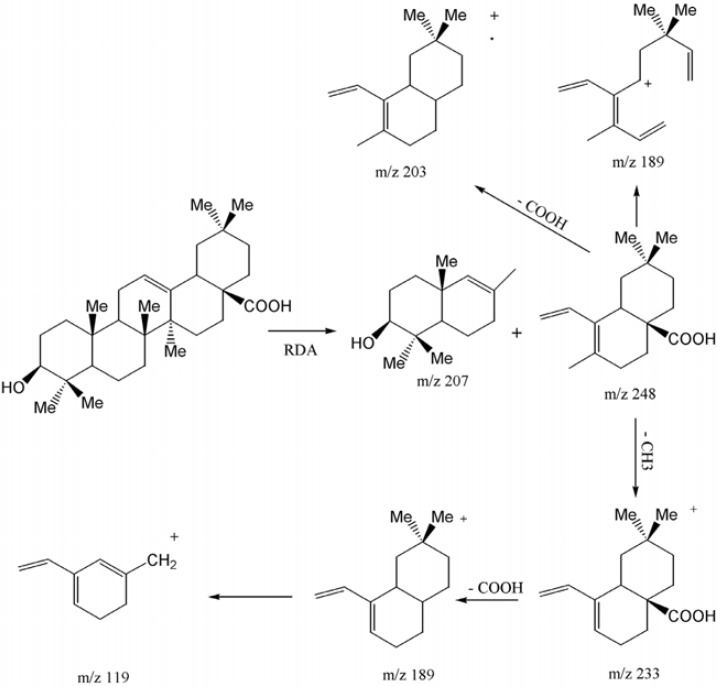
EI-Mass fragmentation pattern of oleanolic acid

**Figure 4:**
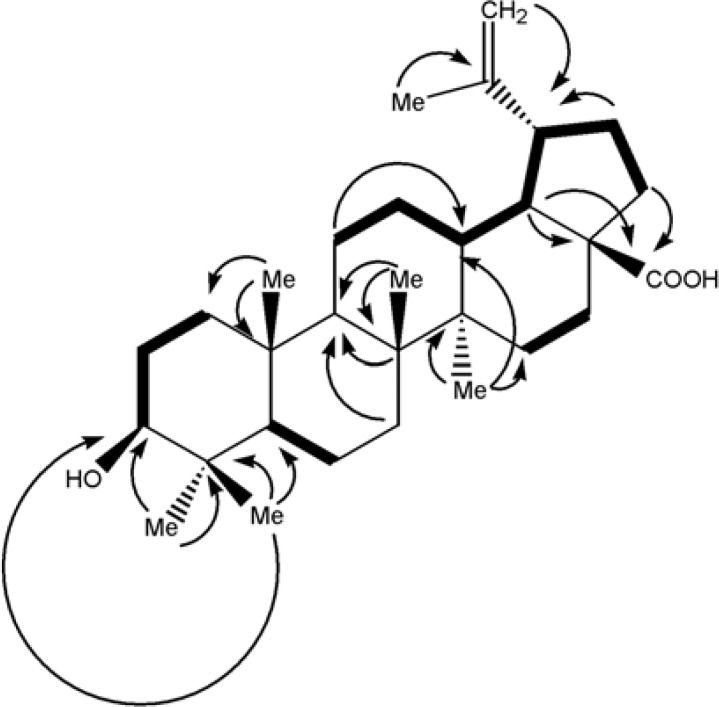
DQF-COSY(in blod) and key (2,3) J(H→C) HMBC cross-peaks in betulinic acid

Compound 2 was obtained as white powder with the positive EI-HRMS of the molecular ion peak at *m/z* 456.3578 indicative of molecular formula of C_30_H_48_O_3_ (calcd. 456.3603) that was in accordance with the number of carbons and hydrogens counted in NMR data (Table 1). The seven degree of unsaturation, ^13^C-NMR and DEPT spectral data (Table 1), suggested five rings plus one acidic carbonyl and an olefinic group in the molecule. The IR spectrum confirmed presence of hydroxyl group (3437 cm^-1^), carbonyl group (1695 cm^-1^) olefinic group C=C (653, 771 cm^-1^) and CO functionalities (1053, 1034 and 1016 cm^-1^). Mass spectrum showed prominent peaks at *m/z* 248 [C_16_H_24_O_2_]^+^ and 207 [C_14_H_23_O]^+^ retro-Diels-Alder (RDA) fragments characteristic for Δ^12^-amyrine series with COOH group (6), 203 [C_15_H_23_]^+^ due to the loss of COOH from *m/z*248 along with other fragments at *m/z* 438 [M - H_2_O]^+^, 410 [M - HCOOH]^+^, 392 [410 - H_2_O]^+^, 189 [C_14_H_21_]^+^ , 175 [C_13_H_19_]^+^, 133 [C_10_H_13_]^+^, 119 [C_9_H_11_]^+^ and 69 [C_5_H_9_]^+^ in Figure 4. The ^13^C-NMR and DEPT spectra confirmed presence of thirty carbon consisted of eight quaternary, five tertiary, ten secondary carbons and seven methyls. ^1^H-NMR showed signals for an olefinic proton at δ_H_ of 5.26 (t, *J *= 3 Hz), a carbinolic proton at δ_H _of 3.20 (dd, *J*_ax,a x_ = 11.5, *J*_ax,eq_ = 4 Hz) suggesting it’s axial and α orientation and δ_H_ of 2.81 (dd, *J *= 13.5, 3.5 Hz) along with seven singlet methyls at δ_H _of 1.23, 1.11, 0.97, 0.91, 0.89, 0.88 and 0.77. All the above data of compound 2 identified it as oleanolic acid (7).

**Figure 5:**
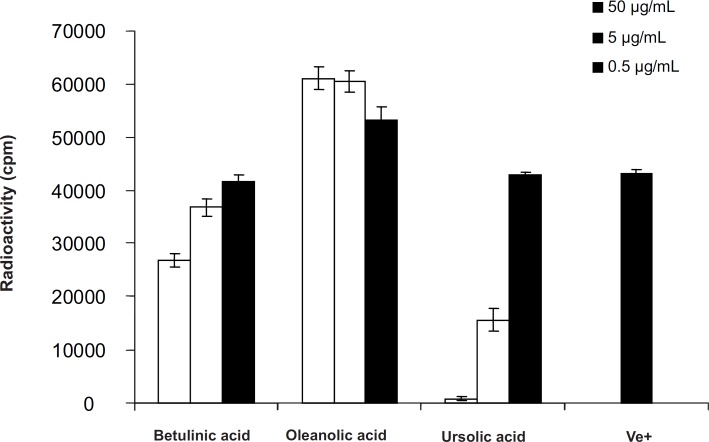
T-Cell Proliferation assay of pentacyclic triterpenes in *Euphorbia microsciadia*

Compound 3 was obtained as white powder with the positive EI-HRMS of the molecular ion peak at *m/z* 456.3622 indicative of molecular formula of C_30_H_48_O_3_ (calcd. 456.3603) that was in accordance with the number of carbons and hydrogens counted in NMR data (Table 1). The seven degrees of unsaturation, ^13^C-NMR and DEPT spectral data (Table 1), suggested five rings plus one acidic carbonyl and one olefinic group in the molecule. The IR spectrum confirmed the presence of hydroxyl group (3442 cm^-1^) and carbonyl group (1710-1690 cm^-1^). Mass spectrum showed peaks at *m/z* 248 as a strong peak and *m/z* 207 in smaller extent as characteristic RDA fragments characteristic for 12-13 double bond *α*- or *β*-amyrine series with COOH group (6) that was confirmed by *m/z* 203 [248 - COOH] as a prominent peak with other fragments at *m/z* 438 [M - H_2_O]^+^, 410 [M - HCOOH]^+^, 189 [C_14_H_21_]^+^, 133, 119 and 55 . ^1^H-NMR spectrum showed an olefinic proton at δ_H_ of 5.27 (t, *J *= 3.3 Hz), a proton geminal to hydroxyl group at δ_H _of 3.19 (dd, *J*_ax,ax_ = 10.8, *J*_ax,eq _ = 5.1 Hz) inferring its *α* - and axial orientation δ_H_ of 2.81 (br-d, *J *= 9 Hz) five singlet methyls at δ_H_ of 1.23, 1.12, 0.97, 0.90 and 0.76 (H-23, 27, 26, 24 and 25), two doublet methyls at δ_H_ of 0.84 (d, *J *= 6.6 Hz, Me_30_) and 0.79 (d, *J *= 6.9 Hz, Me_29_) which were characteristics for ursane skeleton (5). The ^13^C-NMR and DEPT spectra confirmed the presence of thirty carbon consisted of seven quaternary, seven tertiary, nine secondary carbons and seven methyls that were in accordance with ursolic acid reported in the literature (8).

*T-Cell proliferation assay*

The antiproliferative action was tested against peripheral blood T-lymphocytes (PBLs). Addition of betulinic acid (1) and ursolic acid (3) to PHA, stimulated PBLs in the concentration ranges of 0.5, 5 and 50 μg/mLresulted in suppression of T-cell proliferation with IC_50_- value > 50 μg/mL and 3.01 ± 0.47 μg/mL, respectively, while, oleanolic acid (2) stimulated lymphocyte proliferations even at the low concentration of 0.5 μg/mL by 24.54% ± 5.51.

## Conclusion

A large number of pentacyclic triterpenes have been investigated for anti-inflammatory properties. Among these are ursolic acid and the lupane-type triterpenes like betulinic acid. Both of these compounds have been tested in a number of *in-vitro* and *in-vivo*model systems (9-13). Betulinic acid possess moderate inhibitory activity at relatively high concentrations on *in-vitro* proliferation assay using PBLs which was in agreement of other published data (12). Ursolic acid was found to potently inhibit T-cell proliferations while the oleanolic acid showed dose-relative stimulatory effect on lymphocyte proliferation assay agreed with another study by Anamika Khajuria (9) which showed dose dependent immunostimulatory effect on *in-vivo* immune functions in mice. Comparing the results, oleanolic acid and ursolic acid with the same structure differ only in E ring, showing different activities (one inhibitory another stimulatory) on PBL proliferations indicating that the E ring (and not A) could be responsible for these changes. On the other hand, ursolic acid was found to potently inhibit T-cell proliferations more than betulinic acid indicating that the activity of six-member E ring is more than its five member analogue in lupane types. Therefore, according to above findings, the combination of E ring size as well as C-19, C-20 and C-28 positions could be responsible for the differences in biological effects in pentacyclic triterpenes analogues. Hence, making derivatives by modification on these positions like C-28 amino-derivatives, changing the C-20 alkene in betulinic acid derivatives, changing the nucleophilicity and the strength of hydrogen bonding capability and/or acidity at position C-28 are suggested to improve their immunomodulatory activities.
